# ZT Optimization: An Application Focus

**DOI:** 10.3390/ma10030309

**Published:** 2017-03-17

**Authors:** Richard Tuley, Kevin Simpson

**Affiliations:** European Thermodynamics Ltd., 8 Priory Business Park, Wistow, Leicester LE8 0RX, UK; kevin@etdyn.com

**Keywords:** thermoelectric, application, simulation, average ZT, optimization

## Abstract

Significant research has been performed on the challenge of improving thermoelectric materials, with maximum peak figure of merit, ZT, the most common target. We use an approximate thermoelectric material model, matched to real materials, to demonstrate that when an application is known, average ZT is a significantly better optimization target. We quantify this difference with some examples, with one scenario showing that changing the doping to increase peak ZT by 19% can lead to a performance drop of 16%. The importance of average ZT means that the temperature at which the ZT peak occurs should be given similar weight to the value of the peak. An ideal material for an application operates across the maximum peak ZT, otherwise maximum performance occurs when the peak value is reduced in order to improve the peak position.

## 1. Introduction

A significant body of research has focused on developing and optimizing new thermoelectric materials [1,2]. However, commercially available devices are still predominantly based on bismuth telluride alloys. The significant material cost as a fraction of the module cost [3,4], and limited temperature range, has limited the application of bismuth telluride thermoelectric modules, especially for use as thermoelectric generators. Transferring promising new thermoelectric materials into reliable, high performance, low cost, thermoelectric modules must overcome a number of challenges, including reproducibly scaling the material, forming very low resistance joints to the material and forming a module with sufficient mechanical strength, thermal stability and lifetime. Even when significant effort to examine these issues is undertaken, several system demonstrators have found it challenging to produce the initially expected significant performance improvements over bismuth telluride modules. Long-term thermoelectric material research has typically had to occur before detailed examination of any application in a system, so that there is often more limited scope for optimizing the material for a specific system. This has typically led to optimization of the peak figure of merit, ZT, becoming the principal aim of material research. The figure of merit, ZT, is defined as ZT = α^2^σT/κ, where α is the Seebeck coefficient, σ is the electrical conductivity, T is the absolute temperature, and κ is the total thermal conductivity. This has occurred despite the knowledge that average ZT across the temperature range is a better predictor of device performance [2]. This paper considers several potential application scenarios, and examines the impact of optimizing a thermoelectric material’s properties for these applications, linking the system conditions and materials to predict device performance. This paper therefore provides quantified examples of the effect of different material optimization strategies on device performance. The material optimization is first considered for commercial bismuth telluride materials for two application scenarios: an energy harvester, with a module hot side of 50 °C and a cold side of 30 °C; and a higher temperature waste heat source where commercial bismuth telluride modules are used close to their maximum temperature, with the hot side at 250 °C and the cold side at 30 °C.

When using an optimized thermoelectric module in a system, typically only around half the temperature difference available at the system level occurs across the thermoelectric material itself [5,6,7]. This occurs due to the simultaneous need for the module architecture to minimize the electrical resistance while maximizing the temperature difference across the thermoelectric material. This significantly lowers the typical operating temperatures experienced by the thermoelectric material. Therefore, for example, in the significant potential application of thermoelectric generators for automotive exhaust waste heat recovery, although average exhaust gas temperatures for a petrol engine can be 500–600 °C, peaking at 1000 °C [8], the optimized thermoelectric generator will need to be optimized to work at a hot side temperature of closer to 400 °C. The exact hot side temperature to optimize for is dependent on engine, drive cycle, cold side cooling and ZT curve shape, but will still occur significantly below the exhaust gas temperature. This scenario with a cold side at 30 °C is therefore considered with a silicide higher temperature material. 

## 2. Modelling Approach

In order to model how material optimization for a particular application might proceed, an approximate, physically realistic model of the material parameters is needed. A two parabolic band model was used, following Ref [9], considering acoustic phonon scattering only (scattering factor = −1/2). In addition to the doping dependent mobility therein described, the T^−3/2^ temperature dependence of the mobility from acoustic phonon scattering was added [10,11]. For the silicide material, the alloy scattering mobility is also significant, so is included with a T^−1/2^ dependence [10]. A constant activated dopant density was assumed, and this is used with the charge neutrality condition to calculate the Fermi level, and thus the rest of the required parameters. Full details of the equations used are given in the supplementary information. For the bismuth telluride material the lattice thermal conductivity was assumed to obey a 1/T dependence [11]. This achieved a slightly better fit to experimental data than a constant lattice thermal conductivity, but the bismuth telluride’s low lattice thermal conductivity makes results less sensitive to its precise temperature dependence. The higher lattice thermal conductivity and larger temperature range in the silicide material makes its temperature dependence more significant. For the silicide material a linear dependence with temperature was assumed [10,12]. The temperature dependent properties were calculated in Matlab at 5 °C intervals.

Although the full module performance will include effects from the compatibility between the n and p-type materials, and the electrical and thermal contact resistance values, it is difficult to generalize these across different material systems and their doping levels. Therefore, a single n-type thermoelectric leg is considered with perfect contacts. The power output from a leg depends on its optimized geometry, and thus the system, so for the purpose of optimizing the thermoelectric material parameters, the efficiency alone is considered. It can be seen that when considering a system, the material ZT and therefore the module efficiency, rather than the module power at fixed temperatures, can actually be most important for maximizing system power [5,6,13].

The efficiency is calculated by finite element analysis (FEA) in a 1D model in COMSOL Multiphysics using the calculated material parameters from Matlab.

## 3. Bismuth Telluride Based Material

**Figure 1:**
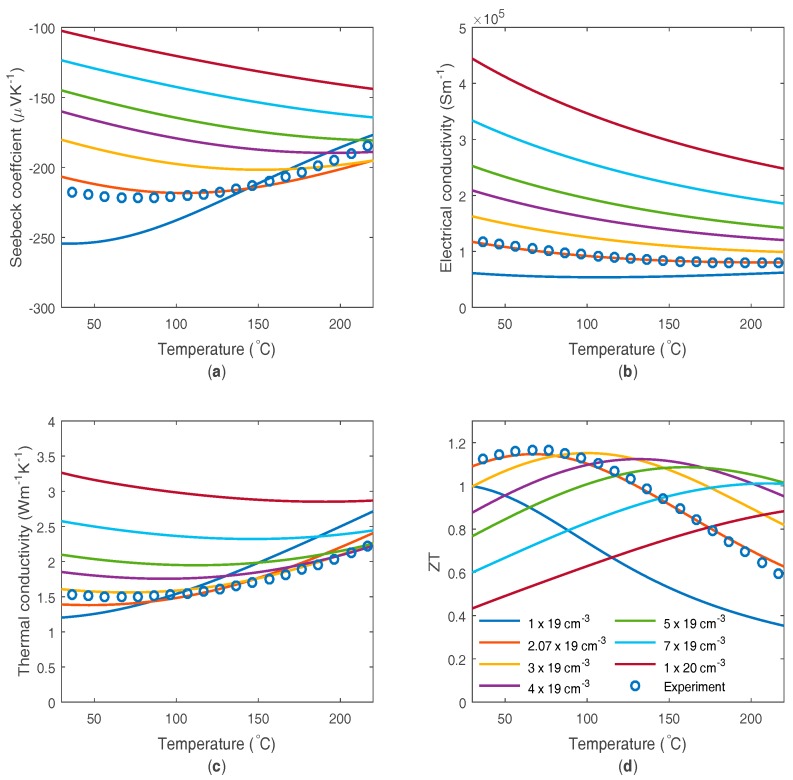
Modelled and experimental thermoelectric material parameters of n-type bismuth telluride as a function of temperature: (**a**) Seebeck coefficient; (**b**) electrical conductivity; (**c**) thermal conductivity and (**d**) ZT. Modelled data includes a number of different doping levels, with the best fit represented by 2.07 × 10^19^ cm^−3^ (orange).

Commercially available bismuth telluride material such as that used in general purpose commercial devices is considered first. The model is fitted against the manufacturer provided data; as shown in Figure 1. The material input parameters are strongly interlinked, so initial parameter choice was guided by the literature on optimized material, and then refined to get the best simultaneous fit to the thermoelectric material properties. Since there exists little literature on minority carrier properties, values obtained for p-type Bismuth telluride were used. The properties used are given in Table 1.

It can be seen that even this relatively simple model, with the inclusion of bipolar effects, can capture much of the thermoelectric characteristics of the material, despite ignoring the effects of non-parabolic bands, temperature dependent band properties and material anisotropy. The bipolar effects are required to produce the peak in Seebeck coefficient and to add to the rise in the thermal conductivity at high temperatures, both of which impact on the ZT curve shape. More complex models are likely to only produce a slightly improved fit to a material like bismuth telluride, but will produce differences upon changes in doping and use of a wider temperature range, and will have more predictive power when considering more major changes in the materials.

Using this model, a number of approximately physically realistic thermoelectric material parameter shapes can be produced, so that they can be used to investigate the impact on performance. For example the doping level can be varied. This assumes that the other properties are unaltered by the doping changes, and that very high doping levels can be reached, which may not always be physically achievable. The changes in thermoelectric materials as the doping level changes is shown in Figure 1. The increase in doping increases the electrical conductivity, while decreasing the Seebeck coefficient and moving its peak to higher temperatures (by reducing the impact of minority carriers). The increase in doping increases the thermal conductivity through the electrical conductivity, but reduces the rise at high temperatures due to the bipolar contribution. The ZT peak moves to higher temperatures on increased doping, with the value at the peak increasing and then decreasing as doping increases. Therefore a maximum peak ZT exists as a function of doping density.

**Figure 2:**
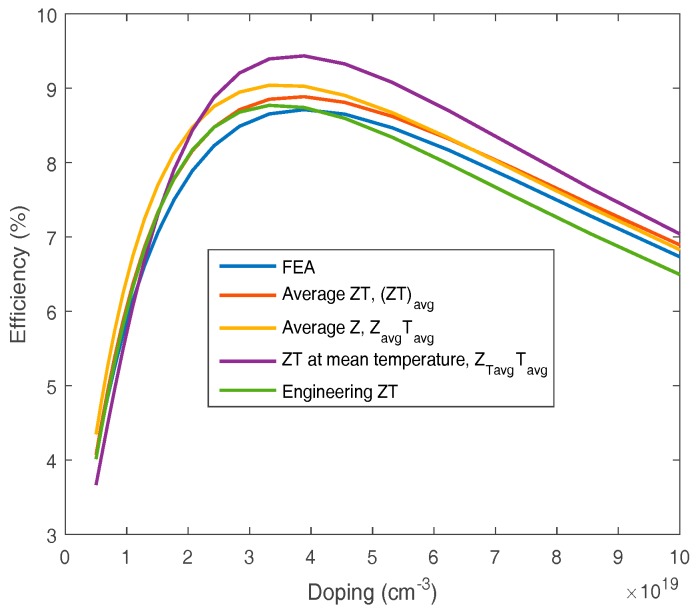
Thermoelectric material efficiency as a function of Bismuth telluride doping density with a hot side temperature of 250 °C and a cold side temperature of 30 °C calculated by different methods.

The FEA efficiency calculation has been compared against simpler commonly used approximations as a function of material doping with a hot side temperature of 250 °C and a cold side temperature of 30 °C. The most common methods convert the temperature dependent material data into a single ZT value by an averaging process which is then used to calculate the efficiency [18]. The single ZT value was calculated either by averaging ZT across the temperature range (ZT)_avg_, averaging Z across the temperature range, Z_avg_T_avg_, or to use the ZT value at the mean temperature, Z_Tavg_T_avg_. The values calculated by these methods, and the efficiency derived from the engineering ZT without the Thomson effect [18], are shown in Figure 2. It can be seen that for these ZT curve shapes using only the ZT value at the mean temperature significantly overestimated the efficiencies. All the other methods give similar results, with the average ZT and engineering ZT method giving the most consistent results with the FEA model.

**Figure 3:**
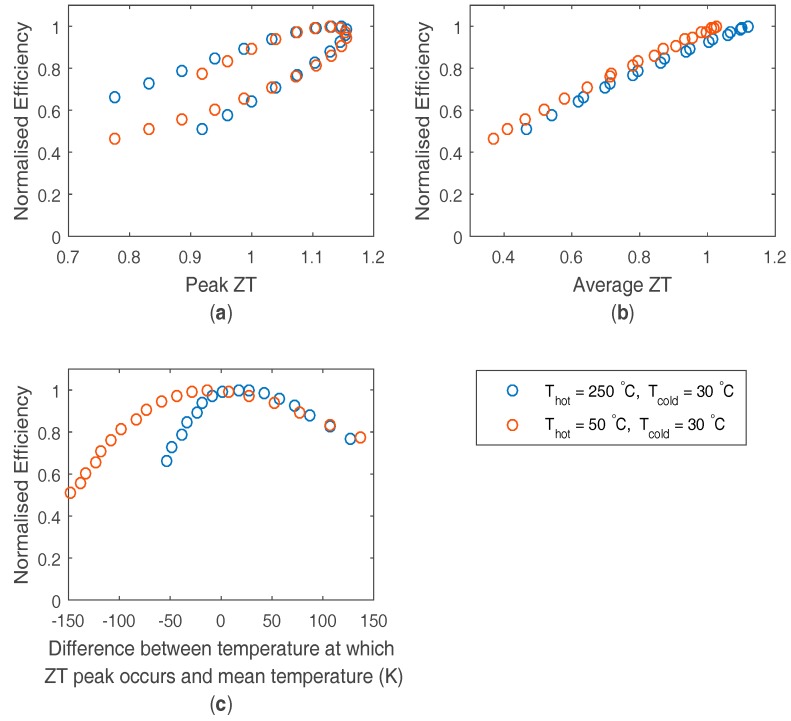
Calculated efficiency by finite element analysis (FEA), normalized to the maximum efficiency for the application, at different material doping levels (from 5 × 10^18^ to 1 × 10^20^ cm^−3^, logarithmically spaced) as a function of (**a**) peak ZT value; (**b**) average ZT value and (**c**) peak ZT position in temperature relative to the mean module temperature. Two different temperature applications are shown.

This model was then used to investigate the link between efficiency and potential material optimization targets under two temperature conditions: an energy harvester, with a module hot side of 50 °C and a cold side of 30 °C; and a higher temperature waste heat source where commercial bismuth telluride modules are used close to their maximum temperature, with a hot side temperature of 250 °C and a cold side of 30 °C. The efficiency in Figure 3 is plotted as a function of three potential optimization targets: the average ZT between the module hot and cold side temperatures, the peak ZT value and the peak ZT position in temperature compared to the mean module temperature. It can be seen that the efficiency is most strongly linked to maximizing the average ZT value. The peak ZT value is significant in achieving this, but the position of the ZT peak is also of similar importance. It is desirable that the peak ZT value occurs close to the module mean temperature to maximize the average ZT so that the module is working across the peak ZT. This demonstrates the advantage of bismuth telluride materials, as under these temperature conditions, both a high peak ZT and good peak ZT position can be simultaneously achieved for the application, resulting in a good average ZT and thus high module performance. It is worth noting that to achieve the peak efficiency some compromise in peak ZT can be worthwhile if it achieves a more favorable peak ZT position.

## 4. Silicide Based Material

**Figure 4:**
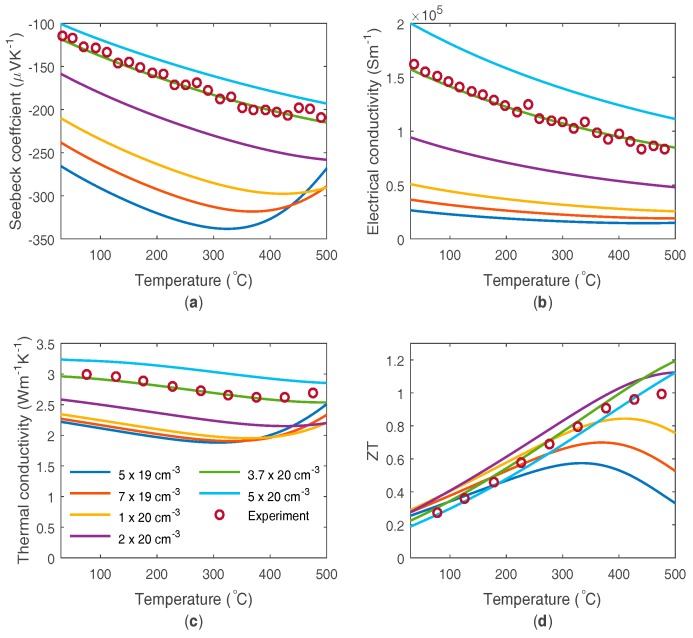
Modelled and experimental thermoelectric material parameters of n-type Sb doped Mg_2_Si_0.4_Sn_0.6_, as a function of temperature: (**a**) Seebeck coefficient; (**b**) electrical conductivity; (**c**) thermal conductivity; and (**d**) ZT. The modelled values are displayed for a number of different doping densities.

An n-type silicide material, Sb doped (0.5–1.5 mol. %) Mg_2_Si_0.4_Sn_0.6,_ is considered for the higher temperature application. Experimental data is taken from Ref. [10], with most of the modelling parameters taken or derived directly from the modelling performed in Ref. [10], with the two different conduction bands combined into a single band. The properties used are given in Table 2, and the good fit to the experimental data is shown in Figure 4. The modelled thermoelectric material parameter’s dependence on doping level is also shown in Figure 4. The lower doping density tends to move the peak ZT to lower temperatures, but below a certain doping level it also reduces the peak ZT value.

**Figure 5:**
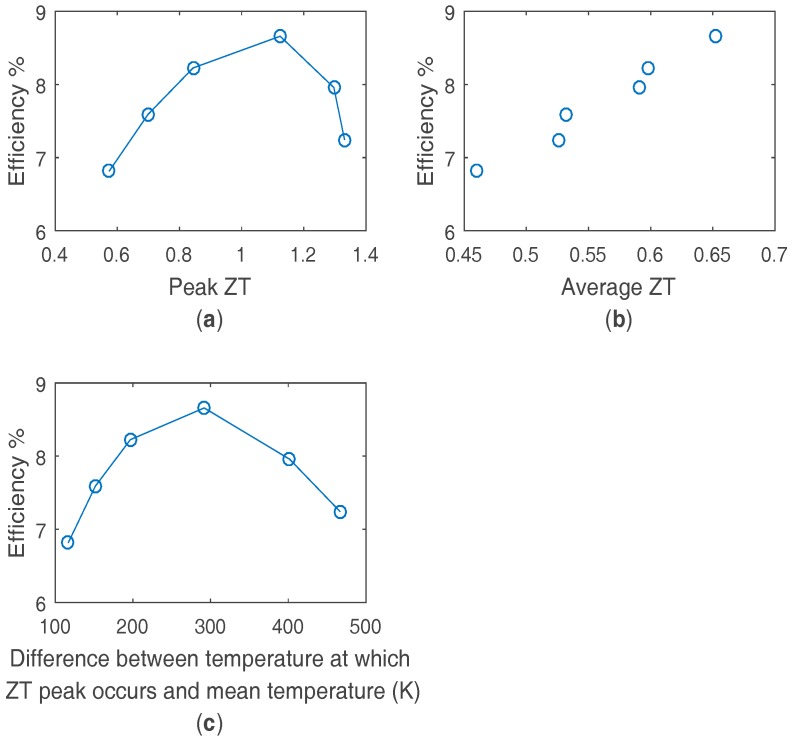
Calculated efficiency by FEA for n-type silicide material, for different material doping levels as a function of (**a**) peak ZT value; (**b**) average ZT value and (**c**) peak ZT position in temperature relative to the mean module temperature. Hot and cold side temperatures are 400 °C and 30 °C respectively.

The impact on efficiency can be calculated for an example automotive application with a hot side of 400 °C and a cold side of 30 °C, and is shown in Figure 5. In a similar manner to the bismuth telluride optimization it can be clearly seen that optimizing for average ZT is preferable to optimizing for peak ZT. Due to the high temperature at which the maximum ZT peak occurs for the silicide material, this effect is stronger than for bismuth telluride applications, so that despite the potential 19% increase in peak ZT, optimizing purely for peak ZT could result in a 16% lower performance than optimizing for the best average ZT. This is due to the better low temperature performance of lower doped, lower peak ZT material. The efficiency is also a strong function of the temperature position of the ZT peak. However the optimum efficiency occurs when the ZT peak occurs at a significantly higher temperature than the mean module temperature, as the high temperature at which the maximum peak ZT occurs biases the balance between peak ZT and peak ZT position to higher temperatures. The high temperature at which the maximum peak ZT occurs compared to the application, and the subsequent lowered average ZT values of the silicide material compared to bismuth telluride material demonstrates the challenge of significantly improving upon the efficiency of bismuth telluride even with the advantage of higher temperature differences. However the higher temperature operation of a material such as a silicide can still significantly improve the power output per module, improving the crucial cost per Watt metric.

## 5. Conclusions

We have used an approximate thermoelectric material model to demonstrate that an average ZT across the temperature range is a good optimization target for thermoelectric material applications. The importance of average ZT means that the temperature at which the ZT peak occurs should be given similar weight to the value of the peak ZT. This means that materials used in applications that allow operation across the peak ZT, such as commonly seen for bismuth telluride, have a significant advantage. Some reduction in peak ZT in order to improve peak position can lead to improved average ZT and thus improved module performance. We have quantified this effect for two materials and three application scenarios, demonstrating that it is possible to increase ZT by 19% while decreasing performance by 16%. However optimizing the material for maximum average ZT does require some knowledge of the targeted application temperatures. Since any thermoelectric module in a system is optimized for maximum power when approximately only half the temperature difference of the system is across the thermoelectric material, many materials (e.g., silicides, skutterudites and Half-Heuslers) have an maximum peak ZT that occurs at temperatures significantly higher than desirable for applications such as automotive exhaust waste heat recovery. Therefore, the extremely challenging but desirable target would be a material which had an maximum peak ZT around 200–250 °C, but was stable to at least 400 °C, and preferably much higher temperatures to allow a suitable module joining process. Alternatively, investigation of methods that can lead to ZT shapes not well described by a simple model, especially flatter ZT curves [19] could result in a significant advantage in a device.

## Figures and Tables

**Table 1:** Properties used to match modelled n-type bismuth telluride material to experimental data.

Property	Used in Model	Literature Reported Values	Ref.
Doping level (cm^−3^)	2.07 × 10^19^	1–5 × 10^19^	[14]
Band gap (eV)	0.16	0.11–0.2	[15,16]
CB DOS effective mass (m_e_)	1.09	0.95–1.9	[16,17]
VB DOS effective mass (m_e_)	1.85	1.5–2.1	[16,17]
Electron mobility at low carrier concentration at 300 K (cm^2^·V^−1^·s^−1^)	389	200–350 ^1^	[16]
Hole mobility at low carrier concentration at 300 K (cm^2^·V^−1^·s^−1^)	138	200–300 ^1^	[16]
Lattic thermal conductivity at 300 K (Wm^−1^·K^−1^)	0.742	0.9–1.6	[15]

^1^ At elevated doping levels.

**Table 2:** Properties used to match modelled n-type silicide material to experimental data.

Property	Value
Doping level	3.7 × 10^20^ cm^−3^
Band gap	0.42 eV
CB DOS effective mass	3.13 m_e_
VB DOS effective mass	1.45 m_e_
Acoustic phonon scattering electron mobility at low carrier concentration at 300 K	135 cm^2^·V^−1^·s^−1^
Alloy scattering electron mobility at low carrier concentration at 300 K	47.3 cm^2^·V^−1^·s^−1^
Acoustic phonon scattering hole mobility at low carrier concentration at 300 K	291 cm^2^·V^−1^·s^−1^
Alloy scattering hole mobility at low carrier concentration at 300 K	102 cm^2^·V^−1^·s^−1^
Lattic thermal conductivity at 300 K	2.1 W·m^−1^·K^−1^
Lattice thermal conductivity gradient	−0.0016 W·m^−1^·K^−2^

## Supplementary Materials

The equations describing the model set-up are available online at www.mdpi.com/1996-1944/10/3/309/s1.
