# Supplementary material for: ZT Optimization: An Application Focus
Source: Materials (Basel). 2017 Mar 17;10(3):309. doi: 10.3390/ma10030309 (PMC5503391; doi:10.3390/ma10030309)
Supplement: Supplementary file 1 [file materials-10-00309-s001.pdf]

Supplementary Materials: ZT Optimization: An Application Focus

Richard Tuley, Kevin Simpson

1. Introduction

The modelling approach was based on the theory found in References [1] and [2]. The equations used are explicitly stated here for completeness.

2. Fermi-Dirac Integrals

The Fermi Dirac integrals used in this supplementary information are defined as:

$$F_j(\epsilon) = \int_0^\infty \frac{E^j}{1+e^{E-\epsilon}} dE. \tag{1}$$

They were numerically solved in Matlab using the script in reference [3] using the numerical approximations referenced within, modified to remove the gamma function.

3. Fermi level

The approach taken was to fix the doping level, N_d, and the material parameters, which can then be used to determine the Fermi level position. Charge neutrality gives the conduction band electron concentration, n and the valence band hole concentration, p, as

$$n = N_d + p \tag{2}$$

as any additional carriers must have been excited across the band gap. From standard band theory

$$n = 4\pi \left(\frac{2m_n^* kT}{h^2}\right)^{\frac{3}{2}} F_{\frac{1}{2}}\left(\frac{E_f}{kT}\right) \tag{3}$$

$$p = 4\pi \left(\frac{2m_p^* kT}{h^2}\right)^{\frac{3}{2}} F_{\frac{1}{2}}\left(\frac{E_g - E_f}{kT}\right) \tag{4}$$

where m_n^* and m_p^* is the density of states effective mass of the conduction band and valence band respectively. E_g is the band gap and E_f is the Fermi level, with the energy scale zeroed at the bottom of the conduction band. By combining Equations (2)–(4), these were numerically solved (using the Matlab function fzero) to find E_f.

The Fermi level can be redefined as the reduced Fermi energy for the conduction band, ϵ_n, and valence band, ϵ_p respectively:

$$\epsilon_n = \frac{E_f}{kT} \tag{5}$$

$$\epsilon_p = \frac{E_f - E_g}{kT} \tag{6}$$

4. Conductivity

The conductivity can be separately calculated for each band as σ_v, where $v = n$ or p respectively for the conduction and valence band:

$$\sigma_v = 2\left(\frac{2\pi m_v^* kT}{h^2}\right)^{\frac{3}{2}} q\mu_{vc} \frac{F_{s+\frac{1}{2}}(\epsilon_v)}{\Gamma(s+\frac{1}{2})} \tag{7}$$

where μ_{vc} is the temperature dependendent mobility in the limit of low carrier concentration (i.e. the conductivity formula has already taken into account the mobility's dependence on doping as $F_0(\epsilon_v)/F_{\frac{1}{2}}(\epsilon_v)$). The scattering factor is taken as $s = -\frac{1}{2}$ in this work, corresponding to acoustic phonon scattering. For acoustic phonon scattering only (used for the bismuth telluride data):

$$\mu_{vc} = \mu_{v0\ acoustic} \left(\frac{T_0}{T}\right)^{\frac{3}{2}} \tag{8}$$

where $\mu_{v0\ acoustic}$ is the known input value of the acoustic phonon scattering limited mobility in the limit of low carrier concentration at a temperature T_0. For the combination of acoustic and alloy scattering used for the silicide material:

$$\frac{1}{\mu_{vc}} = \frac{1}{\mu_{v0\ acoustic} \left(\frac{T_0}{T}\right)^{\frac{3}{2}}} + \frac{1}{\mu_{v0\ alloy} \left(\frac{T_0}{T}\right)^{\frac{1}{2}}} \tag{9}$$

where $\mu_{v0\ alloy}$ is the known input value of the alloy scattering limited mobility in the limit of low carrier concentration at a temperature T_0.

The total conductivity is then simply given by:

$$\sigma = \sigma_n + \sigma_p. \tag{10}$$

5. Seebeck coefficient

The Seebeck contribution for each band ($v = n$ or p) is given by:

$$\alpha_v = \mp\frac{k}{e}(\delta_v - \epsilon_v) \tag{11}$$

$$\delta_v = \frac{(s+\frac{5}{2})F_{s+\frac{3}{2}}(\epsilon_v)}{(s+\frac{3}{2})F_{s+\frac{1}{2}}(\epsilon_v)} \tag{12}$$

where for the conduction band $-$ is used, and $+$ for the valence band. The total Seebeck coefficient, α, is then given by:

$$\alpha = \frac{\alpha_n\sigma_n + \alpha_p\sigma_p}{\sigma_n + \sigma_p}. \tag{13}$$

6. Thermal conductivity

The Lorentz number, L_v, is given by:

$$L_v = \left(\frac{k}{e}\right)^2 \left(\frac{s+\frac{7}{2}}{s+\frac{3}{2}}\frac{F_{s+\frac{5}{2}}(\epsilon_v)}{F_{s+\frac{1}{2}}(\epsilon_v)} - \delta_v^2\right). \tag{14}$$

The total electron and hole contribution to the thermal conductivity is then given by

$$\kappa_{el} = \sigma_n L_v T + \sigma_p L_p T + \frac{\sigma_p\sigma_n}{\sigma_p + \sigma_n}(\alpha_p - \alpha_n)^2 T. \tag{15}$$

The lattice thermal conductivity for bismuth telluride is given by:

$$\kappa_{lat} = \kappa_0 \frac{T_0}{T} \tag{16}$$

where κ_0 is the known value of the lattice thermal conductivity at temperature T_0. The lattice thermal conductivity for the silicide is given by:

$$\kappa_{lat} = \kappa_1 + \kappa_2(T - T_0) \tag{17}$$

where κ_1 is the lattice thermal conductivity at temperature T_0 and κ_2 is the lattice thermal conductivity gradient (a negative value). The total thermal conductivity is then given by

$$\kappa = \kappa_{el} + \kappa_{lat} \tag{18}$$

7. Figure of Merit

The figure of merit is then given by:

$$ZT = \frac{\alpha^2 \sigma T}{\kappa} \tag{19}$$

References

1. Rowe, D.M.; *CRC Handbook of Thermoelectrics*, 1st ed.; CRC press: Boca Raton, FL, USA, 1995; pp. 43-54.
2. Zhang, L.; Xiao, P.; Shi, L.; Henkelman, G.; Goodenough, J.B.; Zhou, J.; Suppressing the bipolar contribution to the thermoelectric properties of Mg2Si0.4Sn0.6 by Ge substitution, *J. Appl. Phys.*, **2015**, *117*, 155103
3. Kim, R.; Lundstom, M. *Notes on Fermi-Dirac Integrals*, 3rd ed.; Available online: http://nanohub.org/resources/5475 (accessed on 30 January 2017)
